# Assessing nurses’ digital competence for public health systems: validation of a brief instrument and factors associated with competence

**DOI:** 10.3389/fpubh.2026.1808439

**Published:** 2026-05-20

**Authors:** Monika Wojcieszko, Mariusz Duplaga

**Affiliations:** Department of Health Promotion and e-Health, Institute of Public Health, Faculty of Health Sciences, Jagiellonian University Medical College, Krakow, Poland

**Keywords:** digital competence, digital competence questionnaire, e-health literacy, nurses, psychometric validation, linear regression

## Abstract

**Background:**

Nurses’ digital competence and e-health literacy are essential for safe use of electronic health records and other clinical information technologies, yet validated measures and context-specific evidence remain limited. This study evaluated the Polish adaptation of the Digital Competence Questionnaire for Nurses and examined correlates of nurses’ perceived digital competence in Poland.

**Methods:**

A national cross-sectional computer-assisted web interviewing survey was conducted among practicing nurses (*N* = 800) in late 2025. The dataset was randomly split for polychoric exploratory factor analysis (*N* = 367) and confirmatory factor analysis with ordinal diagonally weighted least squares estimation (*N* = 433). A multiple linear regression model was fitted for the total score in the full sample.

**Results:**

Exploratory analyses indicated one dominant factor; a two-factor benchmark showed limited separability. Confirmatory analysis supported a predominantly unidimensional solution with good approximate fit for the one-factor model (CFI = 0.992; RMSEA = 0.046; SRMR = 0.047); the two-factor benchmark improved fit modestly but the factors were highly correlated (*r* = 0.885). In regression, higher e-health literacy was strongly associated with higher digital competence [B (SE) = 0.71 (0.05), 95% CI: 0.61 to 0.80]. A higher number of applications used at the workplace was also associated with higher digital competence [B (SE) = 0.22 (0.11), 95% CI: 0.01 to 0.43]. Lower daily internet use (vs > 4 h/day) was associated with lower competence [for <1 h/day: B (SE) = −2.97 (0.61), 95% CI: −4.34 to −1.60], while social media use and most sociodemographic and broad workplace indicators were not independently associated. The model explained 25.9% of variance (*R*^2^ = 0.259).

**Conclusion:**

The Polish adaptation of the Digital Competence Questionnaire for Nurses supports use of a concise total score. Digital competence in nurses was most consistently linked to e-health literacy and everyday internet engagement, suggesting that workforce development should combine operational training with skills in information appraisal and safe use of digital resources.

## 1 Introduction

Digital transformation has become a prominent feature of contemporary health systems, reshaping how care is documented, coordinated, and evaluated. Nurses are among the most intensive users of health information technologies, interacting daily with electronic health records, e-medication processes, decision-support functions, bedside and mobile devices, and digital communication channels across organizations. Evidence indicate that digital technologies can improve nursing care, but also introduce new workflow demands and safety risks when usability, integration, or organizational conditions are inadequate (1–4). Consequently, nursing work increasingly requires not only the ability to use digital tools efficiently, but also adequate information management and the capacity to work safely and ethically with data, digital systems, and patients in healthcare settings where care is delivered with the support of digital systems (5).

From a public health perspective, nurses’ digital competence is directly linked to system resilience and equity. Digital solutions are now of fundamental importance for prevention and health promotion, continuity of care for chronic disease, vaccination programs, and outbreak response, the contexts in which timely data capture, reporting, and communication can influence health outcomes across communities (6, 7). At the same time, expanding digital services can widen disparities if professionals are not prepared to support patients with diverse needs, limited access, or low digital skills. Nurses often act as easily accessible mediators between systems and people, interpreting health information and guiding patients through portals, telehealth, remote monitoring, and digital self-management. This means that nurses’ digital competence is not only important for individual patient care, but also for broader goals such as effective public health actions, helping patients take an active role in their care, and making health services accessible and inclusive for everyone (7).

Despite widespread implementation of clinical information systems, development of digital competence in nursing remains uneven. Standards in nursing informatics emphasize that digital competence should be developed over time through basic training and continuing professional education, and it should be updated as technologies change, including data analytics, automation, and AI-based decision support (8, 9). International competency initiatives have clarified what is expected from nurses in informatics, including managing clinical information, exchanging information within care teams and across systems, handling ethical and legal responsibilities, and understanding how digital systems are implemented, maintained, and updated (10). From a broader perspective, nurses also need the general digital competence expected of all citizens, because they use the same online information environments, communication platforms, and privacy-sensitive digital services in daily life and at work (11). Digital competence is not static. It must keep pace with organizational change, software updates, evolving documentation requirements, and stricter expectations for cybersecurity and data protection.

A consistent theme in the literature is that digital competence is shaped by both individual and contextual factors. Studies examining informatics competency among nurses report associations with education and training exposure, role and responsibility, frequency of technology use, and organizational support (12, 13). Studies performed among Finnish nurses also suggests that age, work setting, and local implementation contexts can relate to perceived competence, potentially reflecting differences in learning opportunities, task demands, and system maturity (14, 15). Experiences with electronic health record implementations show that competence develops step by step through repeated adjustment. Early rollouts can disrupt workflows and increase documentation workload, and later upgrades or optimization efforts may improve how the system fits daily practice, but they still require staff training, time to learn, and learning through everyday use (16). From a sociotechnical perspective, competence cannot be reduced to user skills alone. It emerges from interactions between people, tasks, technologies, organizational structures, and external constraints (5, 17). This has practical implications for designing interventions to strengthen nurses’ digital competence and support digital system use. Training alone may be insufficient unless workload, device availability, interface usability, local implementation facilitators, and supportive leadership are also addressed.

An additional dimension with growing relevance is e-health literacy (eHL), understood as the capability to seek, evaluate, and use digital health information and services (18). While originally conceptualized for consumers, digital health literacy has clear professional implications for nurses who must appraise online information, teach patients how to judge source credibility, and support safe engagement with digital services. Studies show that nurses’ eHL varies and may relate to educational level, professional role, and attitudes toward digital technologies, factors that also can shape broader digital competence and readiness to adopt new tools (19, 20). eHL is widely understood as more than the ability to navigate websites or use digital tools. It also involves judging the reliability of online information, deciding what is relevant to a specific health problem, and using digital services in a way that protects privacy and safety, which is particularly important in information environments where misinformation is common (18, 21). In this sense, nurses’ digital competence and eHL are mutually reinforcing: competence enables effective patient guidance, and patient-facing demands can motivate competence development in practice.

The assessment of nurses’ digital competence is essential for workforce planning, educational quality assurance, and evaluation of digital transformation initiatives. A recent systematic review of instruments used to measure nurses’ informatics competence highlights variability in conceptual definitions and psychometric strength across tools, reinforcing the need for theoretically grounded and well-validated measures (22). Beyond informatics-competency scales and context-specific measures, contemporary approaches increasingly operationalize digital competence as a multidimensional construct that includes knowledge and skills, attitudes, and the capacity to apply digital tools meaningfully in clinical workflows (13, 14). Measurement also needs to be sensitive to setting differences, as competence requirements in hospital wards, primary care, long-term care, and public health nursing may diverge in emphasis (e.g., in relation to documentation intensity, telehealth utilization, surveillance reporting, or patient education tasks) (10, 23).

The Digital Competence Questionnaire for Nurses (DCQfN) was developed as a concise, practice-relevant measure of nurses’ digital competence, conceptualized as a broad construct comprising closely related knowledge and skills, as well as attitudes toward digital technology (24). Cultural adaptation studies, including the Turkish validation, supported the feasibility of using the instrument in different nursing contexts while retaining a comparable two-factor structure and good psychometric performance (25).

The aim of this study was twofold. First, it was to evaluate the psychometric performance of the Polish adaptation of the DCQfN, with particular attention to its internal structure and scoring. Second, it was to examine individual and professional factors associated with nurses’ digital competence in Poland, including eHL, everyday digital engagement, education and training background, and workplace characteristics.

Based on prior research and general expectations about digital competence, we formulated *a priori* hypotheses. We expected eHL to be positively associated with digital competence. We also hypothesized that older age would be related to lower perceived digital competence, while higher educational attainment and more recent participation in training would be linked to higher competence. In addition, we expected more frequent Internet use to be associated with higher digital competence. We anticipated that social media use would show a weaker and less consistent association because time spent on social platforms does not necessarily reflect skills relevant to clinical digital work. Finally, we explored whether workplace context and professional characteristics such as specialization are related to digital competence, without specifying a directional hypothesis due to heterogeneity in local implementation conditions.

## 2 Methods

### 2.1 Survey

A cross-sectional computer-assisted web interviewing (CAWI) survey was conducted among nurses practicing in Poland. The study had a cross-sectional observational design and did not involve any intervention, follow-up, or allocation of participants to comparison groups. Recruitment and data collection were implemented by an external research agency (Fieldstat Sp. z o.o.) (26). The sampling plan used stratification with proportional targets derived from available national workforce statistics in Poland, including sex, age group, and voivodeship (27). Additional quotas were applied to ensure coverage of key workplace characteristics (e.g., type of institution and practice setting). Accordingly, the study used a stratified quota-based sampling approach designed to reflect the national distribution of key demographic and workplace characteristics in the target population.

The survey had closed access: eligible respondents were contacted directly by the research agency and received an online invitation containing study information and a unique, non-transferable survey link; the platform did not permit multiple submissions. Eligible respondents were nurses practicing in Poland. Data collection was conducted over 15 working days (November 25, 2025 to December 15, 2025). Of the contacted invitees, 1,618 declined participation and 2,004 interviews were excluded by the contractor due to ineligibility (*n* = 148), non-completion (*n* = 1,015), failed control questions (*n* = 67), excessively short completion time (*n* = 259), or straightlining response patterns (*n* = 515). Respondents were therefore excluded if they were found to be ineligible by the contractor, did not complete the questionnaire, or failed predefined data-quality checks. In accordance with the survey specification agreed with the contractor, only fully completed questionnaires were to be delivered to the authors. Because partially completed questionnaires were removed prior to data delivery, the final analytic dataset comprised fully completed interviews only (*N* = 800) and contained no item-level missing data. A formal *a priori* power analysis was not performed. The target sample size was determined pragmatically to provide a nationwide dataset of sufficient size for the planned psychometric analyses and multivariable regression modeling. Assuming an expected fraction of 0.50 and a 95% confidence level, a sample of 800 nurses corresponds to an estimated margin of error of about ±3.46 percentage points when accounting for the finite population of approximately 323,600 nurses holding a valid licence to practice in Poland (27).

The study protocol was reviewed and approved by the Jagiellonian University Medical College Bioethical Committee of the Jagiellonian University (Decision No. 1072.6120.115.2022, dated May 25, 2022). Participation was voluntary and anonymous, and no direct identifiers were collected. Informed consent was obtained electronically: respondents indicated consent by confirming willingness to participate and proceeding to the questionnaire. Reporting of the web-based survey procedures was guided by established recommendations for Internet surveys (28).

### 2.2 Questionnaire

The questionnaire comprised 74 single items grouped into thematic blocks. It included two standardized instruments, the DCQfN and the eHealth Literacy Scale (eHEALS), and additional items covering Internet and social media use, use of specialized software at the workplace, workplace characteristics, and professional development (participation in vocational trainings and possession of a nursing specialization). Sociodemographic characteristics were also collected, including age, sex, education level, and place of residence. The survey additionally included items not analyzed in the present paper that assessed attitudes toward the use of artificial intelligence in healthcare and perceived related risks.

### 2.3 Instruments and item blocks

#### 2.3.1 The digital competence questionnaire for nurses (DCQfN)

The Digital Competence Questionnaire for Nurses (DCQfN; originally presented as the Digital Competence Questionnaire for clinical practice nurses) was developed by Golz et al. (24) as a brief, practice-oriented self-report measure of nurses’ digital competence that integrates attitudes toward digital technology with the knowledge and skills needed for its use in clinical work. Item development was preceded by a Delphi-based content validation process that generated an initial 26-item pool with high content validity (29). In the subsequent psychometric validation study, the instrument was refined to 12 items forming two 6-item domains: “Attitude” (e.g., perceived benefits and enjoyment of using digital technology at work) and “Knowledge and skills” (e.g., confidence in using digital technology for information seeking, communication, and handling clinical data). Items are rated on a 5-point Likert agreement scale from 1 (“fully disagree”) to 5 (“fully agree”), with higher scores indicating higher self-perceived digital competence. Scores can be computed for each domain and as an overall indicator (24).

##### 2.3.1.1 Cultural adaption of DCQfN

Permission to translate and culturally adapt the DCQfN for use in Poland was obtained from the instrument developers. The adaptation process followed World Health Organization recommendations for translation and linguistic evaluation of health-related instruments, emphasizing conceptual equivalence and clarity rather than literal word-for-word translation (30).

Two independent forward translations were prepared by native Polish speakers with professional experience relevant to healthcare and digital health. Translators were instructed to use natural, commonly understood wording appropriate for Polish nursing practice and to avoid unnecessary technical jargon. An interdisciplinary expert panel (*n* = 7), representing nursing, medicine, healthcare systems, public health, and other health-related areas, reviewed both forward translations, discussed discrepancies, and produced a single consensus Polish version.

Next, two independent back-translations into English were prepared by translators with high proficiency in both Polish and English who were not involved in the study and were blinded to the original wording. The back-translations were compared with the source version, and any inconsistencies were resolved through panel discussion, leading to minor refinements aimed at improving clarity and preserving the intended meaning of items (30).

Pretesting with cognitive interviewing was then conducted among 15 practicing nurses to assess item comprehension, interpretation of key terms, and suitability of response options. The pilot group consisted entirely of women (mean age 31.9 years, SD = 7.3). Most participants had a university master’s degree (13/15; 86.7%), and the average professional experience in nursing was 7.1 years (SD = 7.1). Nearly half held a nursing specialization certificate (7/15; 46.7%). Participants represented diverse practice contexts, including hospital settings (*n* = 7) and outpatient clinics (*n* = 6); one participant reported diagnostic/laboratory work (*n* = 1), and three reported other settings. Cognitive interviews used structured probing to identify misunderstandings and improve clarity and cultural appropriateness. Feedback was reviewed by the panel and the final Polish version was developed (31). English and corresponding Polish version of the questionnaire are provided in Supplementary Table S1.

#### 2.3.2 The e-health literacy scale (eHEALS)

The eHEALS is a brief 8-item self-report instrument developed by Norman and Skinner (32) to assess perceived eHL, understood as confidence in seeking, evaluating, and using online health information. Items are rated on a 5-point Likert agreement scale (from strongly disagree to strongly agree) and are summed to yield a total score, with higher values indicating higher perceived eHL (range 8–40, when coded 1–5) (32). A Polish adaptation and validation of eHEALS (Pl-eHEALS) was published by Duplaga et al. (33), who tested the scale in two nationally representative Polish samples and reported evidence supporting reliability and construct validity of the Polish version, consistent with its use as a unidimensional total score measure.

#### 2.3.3 Digital technology use and IT systems at work

A separate item block assessed respondents’ everyday use of digital technologies and the extent of software use in clinical work. Time spent on Internet use (for private and professional purposes) and social media use was recorded using an ordered set of daily duration categories ranging from “no use” to “more than 5 hours per day”. In addition, respondents indicated which types of specialized software they use at the workplace (yes/no items), covering domains such as electronic patient documentation, outpatient systems, laboratory and radiology systems, and medication management, among others. For analytic purposes, the number of endorsed software categories was summed to create an indicator of the breadth of workplace program use.

#### 2.3.4 Professional and sociodemographic profile

The participant characteristics section captured key workplace, professional development, and sociodemographic information. Workplace items assessed the primary employment setting, including hospital employment and employment in a private facility. Professional development items covered participation in vocational training, including how recently training had been attended, and whether the respondent held a postgraduate nursing specialty certification (i.e., a formal specialization in a specific nursing field). Sociodemographic items included age, sex, education level, and place of residence.

### 2.4 Statistical analysis

All data management and statistical analyses were conducted in IBM SPSS Statistics v29. Dimensionality analyses (polychoric EFA and CFA) were executed within SPSS using its embedded R integration interface and SPSS extension commands. Polychoric/heterogeneous correlation matrices were computed with SPSSINC HETCOR (34). EFA and ordinal reliability were implemented in R with the psych package (35), and CFA was estimated in lavaan (v0.6–17; CRAN) using an ordinal diagonally weighted least squares estimator (DWLS; theta parameterization) (36). Descriptive statistics were computed for all study variables (means and standard deviations for continuous variables; frequencies and percentages for categorical variables).

#### 2.4.1 Exploratory factor analysis (EFA)

The internal structure of the Digital Competence Questionnaire for Nurses (DCQfN; 12 ordered-categorical items) was evaluated using a split-sample strategy with exploratory factor analysis (EFA) followed by confirmatory factor analysis (CFA) in an independent subsample to reduce capitalization on chance (EFA *N* = 367; CFA *N* = 433) (37, 38). Because response categories are ordinal, dimensionality analyses were based on polychoric associations and estimators suitable for ordered-categorical indicators (39, 40).

EFA was performed on the polychoric correlation matrix using principal axis factoring with an oblique rotation (direct oblimin) to allow correlated factors. Factor retention was guided by the eigenvalue profile (Kaiser criterion), scree inspection, and interpretability, consistent with recommended practice in applied psychometrics (37, 38).

#### 2.4.2 Confirmatory factor analysis (CFA)

CFA was performed on all 12 DCQfN items, treated as ordered-categorical indicators using an ordinal diagonally weighted least squares estimator (DWLS; theta parameterization) (41). CFA evaluated two competing specifications. The first was a parsimonious one-factor model in which all items loaded on a single latent digital competence factor. The second was a theory-informed two-factor benchmark model consistent with the original conceptual structure of the instrument, with items 1–6 loading on one latent factor and items 7–12 loading on a second latent factor (24). In the two-factor model, no cross-loadings were specified and the latent factors were allowed to correlate. As an additional sensitivity analysis, we also fitted a bifactor CFA model with one general factor defined by all 12 items and two orthogonal specific factors corresponding to items 1–6 and 7–12. This model was used to examine whether the common variance across items was predominantly captured by a general factor, despite the conceptual distinction between the two proposed subdomains.

Model fit was evaluated using *χ*^2^, CFI, TLI, RMSEA (90% CI), and SRMR, with approximate-fit interpretation informed by widely used recommendations (42, 43). CFI and TLI values ≥0.95 were interpreted as indicating a good approximate fit, whereas values ≥0.90 were considered acceptable. RMSEA values ≤0.06 were interpreted as indicating good approximate fit, and values ≤0.08 as acceptable fit. SRMR values ≤0.08 were considered acceptable. The two nested models were compared using a scaled *χ*^2^ difference test. As a supplementary analysis, the measurement invariance of the final one-factor model was tested across age tertiles (22–45, 46–56, and 57–66 years) in the full sample using multigroup ordinal CFA with DWLS. Invariance across sex was not examined because the male subgroup was very small and highly imbalanced.

Reliability was additionally evaluated using omega-based coefficients. In the validation subsample, model-based reliability was examined using omega coefficients derived from the bifactor solution, including omega total (*ω*) for the total score and omega hierarchical (ωH) for the proportion of reliable variance attributable to the general factor. We also examined the explained common variance (ECV) from the bifactor solution as an ancillary index of the relative predominance of the general factor. In addition, the omega total for the overall DCQfN score was estimated in the full sample to quantify the reliability of the final total-score representation of the scale.

#### 2.4.3 Regression modeling

To examine correlates of nurses’ overall digital competence, we fitted a multiple linear regression model with the total DCQfN score as the dependent variable (*N* = 800). Predictors included age, sex, place of residence, education level, Internet use intensity, social media use intensity, recency of training participation, hospital employment, employment in a private facility, possession of a nursing specialization, the number of IT programs used at work, and eHL. Due to substantial collinearity between age and the length of professional nursing experience, only age was retained as an indicator of career stage in the regression model, whereas professional experience was excluded to avoid redundancy and unstable coefficient estimates. Internet use intensity and social media use intensity, place of residence and education levels were treated as categorical predictors and entered as sets of indicator (dummy) variables. All predictors were entered simultaneously (Enter method). Dichotomous predictors (e.g., sex, hospital employment, employment in a private facility, and possession of a nursing specialization) were coded as binary indicators, whereas variables with more than two categories (place of residence, education level, professional training recency, Internet use intensity, and social media use intensity) were represented by sets of dummy variables using SPSS default reference-category coding. Model performance was evaluated using R^2^, adjusted R^2^ and the overall F-test. We report unstandardized coefficients (B) with standard errors (SE), standardized coefficients (*β*), and 95% confidence intervals (95% CI).

Assumptions of the linear regression model were evaluated using residual diagnostics. Approximate normality was assessed by inspecting the distribution and histogram and probability plots of standardized residuals, whereas homoscedasticity was assessed by plotting standardized residuals against standardized predicted values. Independence of errors was evaluated using the Durbin-Watson statistic. Collinearity was assessed using tolerance and variance inflation factors (VIF), and no evidence of problematic multicollinearity was observed (Supplementary Table S2).

Potential residual outliers were screened using deleted studentized residuals, with values outside ±3 treated as requiring sensitivity assessment rather than automatic exclusion. Their influence on model estimates was further examined using Cook’s distance and leverage values. Because these observations showed limited influence on the fitted model, they were retained in the primary full sample analysis. In addition, a sensitivity analysis excluding observations with deleted studentized residuals outside the ±3 threshold was conducted to assess the robustness of the findings; the corresponding results are presented in the Supplementary Table S12.

## 3 Results

### 3.1 Characteristics of the study group

The sample comprised 800 nurses. The mean age was 48.93 years (SD = 12.04), and the mean professional experience in nursing was 26.65 years (SD = 11.92). Mean eHL was 31.78 (SD = 4.22), and the mean total DCQfN score was 46.52 (SD = 6.52). On average, respondents reported using 5.58 categories of specialized software in their workplace (SD = 2.07). Most participants were female (*n* = 769; 96.1%) (Table 1). Education levels were predominantly Master’s (*n* = 406; 50.7%) or Bachelor’s (*n* = 203; 25.4%). More than half of the sample lived in cities with >100,000 inhabitants (*n* = 413; 51.7%). More than half also reported holding a nursing specialization certificate (*n* = 425; 53.1%). Regarding vocational training, 53.8% had attended professional training within the last 12 months (*n* = 430). Daily Internet use was most commonly 2–4 h (*n* = 281; 35.1%) or >4 h (*n* = 248; 31.1%), whereas daily social media use was typically >2 h (*n* = 259; 32.3%). Hospital employment was reported by 65.0% of respondents (*n* = 520).

**Table 1:** Participant characteristics.

Variable	Category	%	*n*
Sex	Female	96.1	769
	Male	3.9	31
Education	Secondary or post-secondary non university	19.3	154
	University Bachelors’	25.4	203
	University Master’s	50.7	406
	Doctorate	4.6	37
Place of residence	Rural area	12.5	100
	Urban <10,000 inhabitants	7.2	58
	Urban 10,000–100,000 inhabitants	28.6	229
	Urban 100,000–500,000 inhabitants	38.8	310
	Urban >500,000 inhabitants	12.9	103
Postgraduate nursing specialization certificate	No	53.1	425
	Yes	45.9	375
Recency of professional nursing training	Last 12 months	53.8	430
	13–24 months ago	17.9	143
	25–60 months ago	12.0	96
	More than 60 months ago	16.4	144
Daily internet use	<1 h	13.4	106
	1–2 h	20.6	165
	2–4 h	35.1	281
	>4 h	31.1	248
Daily social media use	<0.5 h (incl. no use)	21.1	169
	0.5–1 h	21.9	175
	1–2 h	24.6	197
	>2 h	32.3	259
Workplace setting	Hospital	65.0	520
	Other	35.0	280
Employment in the private sector	No	64.9	519
	Yes	35.1	281

### 3.2 Exploratory factor analysis

EFA was conducted in the exploratory subsample (*N* = 367). Sampling adequacy was high (KMO = 0.909) and Bartlett’s test confirmed factorability (χ^2^(66) = 1655.547, *p* < 0.001) (Supplementary Table S3). The polychoric correlation matrix indicated consistently positive associations among items (mean inter-item polychoric *r* ≈ 0.398; range 0.262–0.609) (Supplementary Table S4), supporting substantial shared variance and the suitability of common-factor modeling (37).

The eigenvalue spectrum indicated one dominant factor (eigenvalue_1 = 5.409; 45.1% of total variance), whereas the second eigenvalue was approximately 1 (0.991) (Supplementary Table S5; Supplementary Figure S1). Communalities after extraction ranged from 0.303 to 0.702 (see Supplementary Table S6). A two-factor oblique benchmark was examined for comparison; the factors were strongly correlated (|*r*| = 0.665), suggesting limited separability. Overall, the EFA pattern supported a predominantly unidimensional structure appropriate for an overall DCQfN score (Supplementary Table S7).

### 3.3 Confirmatory factor analysis

In the full sample (*N* = 800), omega total for the DCQfN score was 0.889, indicating high internal consistency of the overall scale. CFA was then conducted in the validation subsample (N = 433). The one-factor model showed good approximate fit: χ^2^(54) = 103.752 (*p* < 0.001), CFI = 0.992, TLI = 0.990, RMSEA = 0.046 (90% CI 0.033–0.059), SRMR = 0.047 (Table 2). Standardized loadings ranged from 0.527 to 0.712 (Supplementary Table S8; Supplementary Figure S2). The two-factor benchmark improved fit modestly: *χ*^2^(53) = 81.804 (*p* = 0.007), CFI = 0.995, TLI = 0.994, RMSEA = 0.035 (90% CI 0.019–0.050), SRMR = 0.043 (Table 2); however, the latent factors were highly correlated (*r* = 0.885) indicating substantial overlap between the proposed subdomains. Although scaled *χ*^2^ difference test favored the two-factor benchmark (Δ*χ*^2^ = 18.717, Δdf = 1, *p* ≈ 1.5 × 10^−5^) (Table 2), the absolute gains in approximate fit were small.

**Table 2:** CFA fit indices (ordinal DWLS).

Fit index	1-factor model	2-factor model	Bi-factor model
N	433	433	433
χ^2^ (df)	103.752 (54)	81.804 (53)	39.364 (42)
p	<0.001	0.007	0.587
CFI	0.992	0.995	1.000
TLI	0.990	0.994	1.000
RMSEA (90% CI)	0.046 (0.033–0.059)	0.035 (0.019–0.050)	0 (0–0.030)
SRMR	0.047	0.043	0.031
Interfactor correlation	—	0.885	—
Scaled *χ*^2^ difference test (2-factor vs. 1-factor)	Δχ^2^ = 18.717; Δdf = 1; *p* ≈ 1.5 × 10^−5^		Not applicable

This table retains the fit-index format used in the main manuscript. Detailed scaled/robust fit indices and ancillary bifactor indices are provided in Supplementary Tables S9, S10.

As an additional sensitivity analysis, a bifactor CFA was fitted with one general factor defined by all 12 items and two orthogonal specific factors corresponding to items 1–6 and 7–12. This model showed the best global fit, χ^2^(42) = 39.364 (*p* = 0.587), CFI = 1.000, TLI = 1.000, RMSEA<0.001 (90% CI 0–0.030), SRMR = 0.031 (Table 2; see also Supplementary Table S9 for scaled and robust fit indices). In the bifactor solution, loadings on the general factor remained substantial across all items (0.538–0.714), whereas the specific factors were weaker, particularly for the Attitudes domain, where one loading was non-significant and several residual loadings were negative (Supplementary Table S8). Ancillary bifactor indices further supported the predominance of the general factor (ECV = 0.824; Supplementary Table S10). Model-based reliability coefficients derived from the bifactor solution were high. Omega total for the DCQfN total score was 0.898, and omega hierarchical was 0.849, indicating that most reliable variance in the total score was attributable to the general factor rather than to residual specific factors (Supplementary Table S10).

As a supplementary analysis, measurement invariance of the final one-factor model was examined across age tertiles in the full sample. The multigroup results indicated that the model operated comparably across age groups, with no meaningful loss of fit after constraining thresholds and then factor loadings to equality. These findings support the comparability of the DCQfN measurement structure across age tertiles (Supplementary Table S11). Taken together with the EFA results, these findings support the interpretation of the DCQfN as essentially unidimensional, justify the practical use of a single overall DCQfN score, and indicate that the one-factor measurement model is comparable across age groups.

### 3.4 Linear regression of DCQfN

The regression model was statistically significant, *F*(23, 776) = 11.80, *p* < 0.001, explaining 25.9% of variance in the total DCQfN score (*R*^2^ = 0.259; adjusted *R*^2^ = 0.237). The standard error of the estimate was 5.69, and the Durbin–Watson statistic was 1.98. Visual inspection of residual diagnostics did not indicate major departures from approximate normality or homoscedasticity. Collinearity statistics indicated no problematic multicollinearity among the predictors (Supplementary Table S2).

Table 3 presents regression coefficients. After adjustment for all covariates, higher eHL was associated with higher DCQfN scores [B (SE) = 0.71 (0.05), 95% CI: 0.61 to 0.80, Table 3]. A higher number of applications used at the workplace was also associated with higher DCQfN scores [B (SE) = 0.22 (0.11), 95% CI: 0.01 to 0.43]. Lower intensities of Internet use compared to the reference category (>4 h daily) were associated with lower DCQfN (for example, for daily use <1 h, B(SE) = −2.97 (0.61), 95%CI: −4.34 to −1.60). Other predictors, including social media use intensity, were not statistically significant in the fully adjusted model (Table 3). Eleven observations had studentized deleted residuals outside the ±3 threshold. These cases were retained in the primary model and were not interpreted as invalid responses. A sensitivity analysis excluding these observations yielded a broadly similar pattern of findings (Supplementary Table S12), supporting the robustness of the main conclusions while indicating that smaller predictor-level associations should be interpreted cautiously. In that analysis, eHL remained the strongest positive predictor, whereas the association for the number of applications used at the workplace was attenuated and no longer statistically significant.

**Table 3:** Multiple linear regression predicting the total DCQfN score.

Variable	Category	B (SE)	*ß*	95% CI	*p*
e-Health literacy		0.71 (0.05)	0.46	0.61–0.80	<0.001
The number of programs used at work		0.22 (0.11)	0.07	0.01–0.43	0.040
Age (years)		−0.01 (0.02)	−0.02	−0.05 to 0.03	0.637
Sex	Female#				
	Male	−1.89 (1.06)	−0.06	−3.98 to 0.19	0.075
Specialization	No#				
	Yes	−0.73 (0.43)	−0.06	−1.58 to 0.12	0.093
Hospital employment	No#				
	Yes	−0.58 (0.49)	−0.04	−1.55 to 0.39	0.243
Employment in a private facility	No#				
	Yes	−0.55 (0.46)	−0.04	−1.44 to 0.35	0.231
Training recency	Last 12 months#				
	>5 years ago	−0.34 (0.73)	−0.02	−1.77 to 1.09	0.642
	2–5 years ago	−0.25 (0.57)	−0.01	−1.36 to 0.86	0.655
	13–24 months ago	−0.24 (0.56)	−0.01	−1.34 to 0.86	0.664
Place of residence	Urban 100,000–500,000#				
	Rural	−0.31 (0.67)	−0.02	−1.62 to 1.01	0.648
	Urban <10,000	0.26 (0.83)	0.01	−1.38 to 1.89	0.756
	Urban 10,000–100,000	−0.25 (0.50)	−0.02	−1.24 to 0.73	0.616
	Urban >500,000	−0.76 (0.65)	−0.04	−2.05 to 0.52	0.243
Education level	University Bachelors’#				
	Secondary or post-secondary non-university	−0.14 (0.65)	−0.01	−1.42 to 1.14	0.833
	University Masters’	0.24 (0.50)	0.02	−0.75 to 1.23	0.633
	PhD	0.76 (1.04)	0.02	−1.27 to 2.79	0.465
Daily Internet use	>4 h#				
	<1 h	−2.97 (0.70)	−0.15	−4.34 to −1.60	<0.001
	1 to 2 h	−2.00 (0.61)	−0.12	−3.20 to −0.80	0.001
	> 2 to 4 h	−1.18 (0.52)	−0.09	−2.20 to −0.16	0.023
Daily social media use	1 to 2 h#				
	<0.5 h	−0.47 (0.62)	−0.03	−1.69 to 0.75	0.448
	0.5 to 1	−0.51 (0.60)	−0.03	−1.68 to 0.67	0.395
	> 2 h	−0.77 (0.56)	−0.06	−1.86 to 0.32	0.166

Dependent variable: total DCQfN score. Model summary: *R*^2^ = 0.259, adjusted *R*^2^ = 0.237, F(23, 776) = 11.80, *p* < 0.001, # - reference category.

## 4 Discussion

### 4.1 Summary of findings

This cross-sectional CAWI survey of nurses practising in Poland combined two complementary aims. First, we evaluated the internal structure of the Polish adaptation of the Digital Competence Questionnaire for Nurses (DCQfN) and assessed whether the instrument supports the use of a single total score. Second, we examined correlates of the overall DCQfN score, with particular attention to eHL and everyday digital engagement. The main findings can be summarized as follows. The factor analytic evidence supported a predominantly unidimensional structure. This conclusion was based on converging indicators rather than on a single decision rule: EFA showed a clearly dominant first factor, the second eigenvalue was approximately 1, the one factor CFA model showed good approximate fit, and the two-factor benchmark yielded only limited improvement in fit while showing a very high interfactor correlation. Additional support came from the bifactor CFA, in which most reliable variance was attributable to the general factor, and from the supplementary age-group invariance analysis, which indicated comparable measurement properties across age tertiles. In combination, these finding suggest that the proposed subdomains are not sufficiently distinct to provide substantial added value over a parsimonious tool-score representation in the present sample.

In the regression model, eHL was a significant correlate of perceived digital competence, and lower daily Internet use was consistently related to lower DCQfN scores compared with the highest use category. A higher number of IT programs used at work was also positively associated with DCQfN score in the primary model, although this association was attenuated and no longer statistically significant in the sensitivity analysis. In contrast, most sociodemographic indicators, broad workplace characteristics, and training recency showed little independent association with DCQfN and social media use intensity was not independently associated with the outcome.

In the Polish sample, the mean DCQfN total score was 46.52 (SD = 6.52), equivalent to 3.88/5 per item. Because validated versions may differ in item count and composition, cross-study comparisons are most interpretable when expressed as per-item means (or standardized scores). Using this approach, the original validation study suggests higher self-perceived digital competence (4.27/5; estimated from published item means for the final 12-item version, i.e., ≈51.24/60) than in the present sample (24). The Turkish adaptation (17 items) yielded a closely comparable item-level mean (3.87/5; 65.80/85) (25). A recent preprint reporting the results of the study among ICU nurses using the 12-item version reported a mean of 48.58 (SD = 7.02), corresponding to 4.08/5 per item, indicating slightly higher self-perceived digital competence in that setting (44).

### 4.2 e-Health literacy

The regression results indicate that eHL is significantly related to perceived digital competence among nurses. This association remained robust after adjustment for workplace characteristics, demographic variables, and indicators of everyday digital engagement. It also remained the strongest predictor in the sensitivity analysis, excluding observations with large deleted studentized residuals, supporting the stability of this finding. The finding is theoretically expected because eHL captures more than the ability to use digital tools. It reflects perceived capacity to find, understand and use online health information in practice (18). More recent models further stress that eHL also involves judging the credibility and relevance of information, calibrating trust in sources and applying information effectively in clinical decisions and patient communication (45). The DCQfN similarly includes competence related to information seeking, using clinical information, drawing conclusions based on digitally obtained information, and managing confidentiality. Therefore, both constructs share an important informational core. The magnitude of the association suggests that eHL is an important correlate of perceived digital competence and may be relevant to educational or implementation strategies.

A further interpretation is that eHL may capture a broader self-efficacy component related to digital health tasks. Both eHEALS and DCQfN are self-report measures, their association may partly reflect shared variance in confidence and perceived competence rather than only differences in objective skills. Self-efficacy is a known determinant of engagement and learning in technology contexts (46). Nurses who believe they can evaluate online health information may also report greater confidence in using electronic documentation systems, communicating via digital channels, and maintaining confidentiality. This does not reduce the importance of the association; rather, it indicates that interventions may need to address both skill acquisition and confidence building. Training that supports structured practice with feedback may help strengthen both procedural competence and perceived efficacy.

### 4.3 General internet use intensity and social media time

Daily Internet use intensity was associated with DCQfN in a consistent graded way. Compared with the highest use group, lower use categories were associated with lower digital competence scores. This pattern was also retained in the sensitivity analysis, which supports the robustness of this association. Such a finding is consistent with the interpretation that more frequent engagement with digital environments co-occurs with greater procedural fluency, familiarity with interfaces, and confidence in troubleshooting; however, the cross-sectional design does not allow causal inference Such competencies can transfer to workplace systems, even if the content of online activities differs from clinical tasks. Frequent use may also be associated with lower anxiety about updates and errors and with greater willingness to explore new digital functions. In practical terms, Internet engagement may serve as a proxy for everyday digital practice that complements more specialized eHL.

In contrast, social media use intensity was not independently associated with DCQfN after adjustment. This null result should be treated as a substantive finding. The lack of association was also unchanged in the sensitivity analysis. Time spent on social media reflects a wide range of activities and may not be a good proxy for competence relevant to clinical digital work. Social media can involve passive consumption and entertainment-oriented behavior that requires limited information management, structured documentation, or privacy focused workflows. Conversely, professional social media use may support learning and networking, but this might represent only a subset of users. Therefore, a simple intensity indicator may be a weak surrogate for competence relevant digital behaviors. Future studies could distinguish professional and nonprofessional social media use, and could include measures of specific digital activities such as participation in online training, use of evidence resources, or engagement with professional platforms.

### 4.4 Sociodemographic variables and workplace categories

One of the notable findings is the lack of independent association between DCQfN and indicators in the primary model, including education level, place of residence, and age. Sex was not associated with DCQfN in the primary model, but because its coefficient changed in the sensitivity analysis, any sex-related difference should be interpreted cautiously. Broad workplace categories were also not independently associated with DCQfN. In general population studies, digital skills often show gradients by education and urbanization, and these patterns are sometimes described as a second-level digital divide differences in skills rather than access (47, 48). Our results suggest that within a professional group such gradients may be reduced. Nursing is a regulated profession with standardized role requirements and increasing exposure to electronic systems across settings. Digital transformation in health care has made digital interaction unavoidable in many tasks, which may contribute to smaller differences associated with background characteristics. Moreover, the CAWI method requires at least basic digital access and engagement, which can attenuate differences by excluding individuals with very low digital participation (28). In this context, the absence of clear sociodemographic gradients should be interpreted as being more consistent with the interpretation that professional and organizational factors may play a larger role than simple background variables.

In our dataset, age likely captures part of professional experience due to typical career patterns. The lack of an age gradient can reflect counterbalancing mechanisms. Younger nurses may have higher general digital fluency, but older nurses may compensate through work strategies, familiarity with clinical information needs, and stable routines. Moreover, digital systems in health care have expanded over many years, meaning that nurses across age groups have had to adapt. A total self-report score may therefore show limited differences by age when adaptation has occurred widely. This interpretation is also consistent with the supplementary age-group invariance analysis, which supported comparability of the one-factor DCQfN measurement model across age tertiles.

### 4.5 Workplace categories

Similarly, broad workplace categories such as hospital versus non-hospital and public versus private were not associated with DCQfN. This does not imply that the workplace context is irrelevant. Instead, it suggests that broad categories may be too coarse to capture the aspects of the digital environment that shape competence. Sociotechnical models emphasize that outcomes of health information technology depend on implementation quality, usability, workflow integration, training, leadership, and availability of support, rather than on the type of institution alone (5). Two hospitals can differ substantially in system maturity, interoperability, and user support. Some primary care settings may have advanced digital workflows, while some hospitals may still experience fragmented systems and workarounds. Therefore, future analyses should incorporate more direct indicators of organizational digital maturity and system usability. Such measures could include the extent of electronic documentation, perceived usability, availability of informatics support, and opportunities for hands on training.

### 4.6 Continuing education training recency

In this survey, “training recency” captured general continuing professional development in nursing practice rather than informatics- or digital-specific education. Consequently, time since the last course may be too coarse to reflect learning opportunities that directly translate into the skills and attitudes assessed by the DCQfN. Reviews suggest that nurses’ digital competence improves most reliably after focused, hands-on training that is directly linked to workplace practice and supported by the organization, rather than after general continuing education that is not tailored to digital tasks (49, 50). Consistent with this, studies on nursing informatics competence indicate that competence levels are higher when nurses receive structured, system-specific onboarding and when training is organized and supported at the institutional level (14, 51). The absence of an independent association for training recency in both the primary and sensitivity models is consistent with this interpretation. Future studies should therefore distinguish digital-focused education from general continuing professional development and measure training content, dose, and perceived applicability to everyday digital workflows.

Similarly, holding a nursing specialization certificate was not independently associated with DCQfN score. This suggests that specialization status, as measured here, should not be treated as a proxy for digital competence. One plausible explanation is that specialization pathways primarily emphasize domain-specific clinical knowledge and procedures, whereas digital competence and nursing informatics require explicit, practice-oriented exposure to digital workflows and local systems (49, 50). In addition, specialization is heterogeneous across fields and may not map closely onto the digital tasks covered by the DCQfN. Future studies should therefore assess whether the type and content of specialization, particularly the presence of informatics-related components, are associated with perceived digital competence.

### 4.7 Number of software categories used at work

The number of digital functionalities used at work was positively associated with DCQfN score in the primary model, although this association was attenuated and no longer statistically significant in the sensitivity analysis. This suggests a modest and non-robust relationship rather than a stable independent effect. Broader exposure to multiple functions within an organization’s information environment may reflect both greater familiarity with digital workflows and greater workflow complexity, particularly when functions are implemented inconsistently across modules or require frequent context switching. In addition, access to specific functionalities and the expectation to use them are largely shaped by role, ward-level routines, and local implementation choices, so “functionality count” is not a pure marker of individual capability. Limited interoperability and workflow fragmentation within and between clinical information system components have been associated with higher workload and safety risks (5). These findings suggest that perceived digital competence may be shaped not only by individual skills but also by the extent and quality of routine contact with digital systems and how well functionalities are integrated into usable, coherent workflows. Future studies should therefore distinguish the breadth of software exposure from usability, interoperability, and task-specific intensity of use.

### 4.8 Implications for practice and policy

The findings have several implications. The psychometric evidence supports using the DCQfN total score as a concise outcome for monitoring nurses’ digital competence in workforce studies and for evaluating interventions. The bifactor results and the supplementary age-group invariance analysis further support interpreting the DCQfN primarily as a measure of general perceived digital competence rather than as a set of clearly separable subdomains. The observed association with eHL suggests that programmes addressing digital competence may benefit from covering not only operational use of specific systems, but also information appraisal and safe use of digital resources in practice. This interpretation is consistent with European frameworks that conceptualize digital competence as encompassing information and data literacy, communication, and safety (11), and with nursing informatics frameworks that emphasize privacy, data management, documentation quality, and decision support as core competencies (8, 52–54). At the same time, the weak role of sociodemographic variables suggests that workforce development may be more usefully oriented toward modifiable work-related and competence-related conditions, such as opportunities everyday digital practice, and the usability and integration of clinical systems, rather than toward demographic targeting alone. The sensitivity analysis also indicates that the most robust implications concern eHL and general Internet use, whereas smaller predictor-level associations should be interpreted more cautiously. As digital transformation accelerates and AI-supported tools become more common, these findings may help inform workforce planning and the prioritization of training and implementation support (6, 7).

### 4.9 Limitations

Several limitations should be considered when interpreting these results. The study is cross- sectional, so causal relationships cannot be inferred, and the direction of association between eHL and perceived digital competence cannot be established.

All key variables were assessed using self-report instruments. Consequently, the results may reflect perceived confidence, self-efficacy, and response tendencies in addition to actual skills or objective performance. Because both the DCQfN and eHEALS are self-reported, their association may also be partly influenced by shared method variance rather than only by a substantive overlap between the constructs.

Although the CAWI design enabled efficient nationwide data collection, it may also have introduced selection bias. Participation required online access, a willingness to respond in a web-based format, and at least basic digital engagement. Therefore, nurses with the lowest levels of digital engagement may have been underrepresented, which may have reduced variability in digital competence and attenuated associations with background variables, particularly those related to sociodemographic gradients.

Finally, the regression model explained a substantial but incomplete proportion of variance, indicating that additional individual and organizational determinants remain unmeasured. Although the sensitivity analysis showed a broadly similar overall pattern of results, some smaller predictor-level associations were not fully stable after exclusion of outlying cases and should therefore be interpreted cautiously. The flagged observations were retained in the primary model and should not be interpreted as invalid responses. They may instead reflect real heterogeneity in nurses’ digital competence that was not fully captured by the available predictors, including unmeasured differences in workplace digital maturity, exposure to electronic systems, digitally advanced clinical tasks, or reliance on paper-based workflows. Because the study did not directly assess nursing informatics specialization or institution-level digital infrastructure, these explanations remain hypotheses rather than verified characteristics of the flagged observations.

Future research should therefore incorporate more direct indicators of digital competence, such as task-based performance assessment or system use data, together with more detailed measures of organizational context, including institutional digital maturity, system usability, implementation support, technical support, and local training resources. Such studies would benefit from longitudinal and, where appropriate, multilevel modeling that separates individual-level from institution-level influences. However, the present dataset was not designed to support robust institution-level clustering analyses, which limited the possibility of examining contextual effects directly. This would help clarify whether between-setting differences in digital environments account for variance not captured by broad workplace categories alone and whether changes in eHL and digital practice are followed by changes in perceived digital competence over time. In addition, the supplementary bifactor and age-group invariance analyses were conducted within a single national sample, so further validation in other settings and subgroups is warranted.

## 5 Conclusion

In a national sample of nurses, the Polish adaptation of DCQfN demonstrated strong psychometric support for a predominantly unidimensional structure and can be used as a total score to quantify perceived digital competence. The overall CFA pattern, further supported by the bifactor results, indicates that the scale can be treated as essentially unidimensional for applied use. Digital competence was strongly related to eHL and to general Internet use intensity, while many sociodemographic indicators, broad workplace characteristics, training recency, and social media use intensity showed limited independent association. The positive association observed for the number of IT programs used at work in the primary model was attenuated in the sensitivity analysis and should therefore be interpreted with caution. These results identify eHL and general Internet involvement as important correlates of digital competence and suggest that workforce development strategies may usefully address not only operational digital skills, but also information appraisal and safe information practices, alongside organizational conditions that enable effective use of clinical information systems.

## Data Availability

The datasets presented in this study can be found in online repositories. The names of the repository/repositories and accession number(s) can be found at: Zenodo - doi: 10.5281/zenodo.18566680; https://zenodo.org/records/18566680.
