# Supplementary material for: Assessing nurses’ digital competence for public health systems: validation of a brief instrument and factors associated with competence
Source: Front Public Health. 2026 May 20;14:1808439. doi: 10.3389/fpubh.2026.1808439 (PMC13230094; doi:10.3389/fpubh.2026.1808439)
Supplement: Supplementary file 1 [file Data_Sheet_1.pdf]

Supplementary Material

Table S1. English and Polish version of the Digital Competence Questionnaire for Nurses

Item	Item content (English)	Item content (Polish)
Item 1	Digital technology fits well with the way I like to work.	Technologia cyfrowa dobrze wpisuje się w sposób, w jaki lubię pracować
Item 2	I enjoy using digital technology at my workplace.	Lubię korzystać z technologii cyfrowych w moim miejscu pracy
Item 3	I like to use digital technology at work.	Chętnie używam technologii cyfrowych w pracy
Item 4	I believe that digital technology provides numerous benefits in terms of quality of care.	Uważam, że technologia cyfrowa zapewnia wiele korzyści w zakresie jakości opieki
Item 5	I believe that digital technology improves patient outcomes.	Uważam, że technologia cyfrowa poprawia wyniki leczenia pacjentów
Item 6	I believe that digital technology is beneficial for my patients.	Wierzę, że technologia cyfrowa jest korzystna dla moich pacjentów
Item 7	I am familiar with the digital technologies at my workplace.	Technologie cyfrowe w moim miejscu pracy są mi znane
Item 8	I feel confident about using digital technology to find relevant information.	Czuję się pewnie, korzystając z technologii cyfrowej w celu znalezienia odpowiednich informacji
Item 9	I feel confident about using digital technology to communicate.	Czuję się pewnie, jeśli chodzi o korzystanie z technologii cyfrowej do komunikacji
Item 10	I feel confident about using digital technology to obtain data and information on clinical care.	Czuję się pewnie, jeśli chodzi o korzystanie z technologii cyfrowej w celu uzyskania danych i informacji na temat opieki klinicznej
Item 11	I am able to reach conclusions based on information acquired through digital technologies.	Jestem w stanie wyciągać wnioski na podstawie informacji uzyskanych za pomocą technologii cyfrowych
Item 12	I feel confident in dealing with confidentiality issues relating to digital technology at my workplace.	Czuję się pewnie, jeśli chodzi o kwestie poufności związane z technologią cyfrową w moim miejscu pracy

Table S2. Collinearity diagnostics (tolerance and VIF)

Variable	Category	Tolerance	VIF
e-Health literacy		0.97	1.03
The number of programs used at work		0.85	1.18
Age (years)		0.79	1.27
Sex		0.96	1.04
Specialization		0.87	1.15

Hospital employment		0.73	1.37
Employment in a private facility		0.86	1.16
Training recency	>5 years ago	0.82	1.22
	2-5 years ago	0.86	1.17
	13-24 months ago	0.88	1.14
Place of residence	rural	0.82	1.22
	urban <10,000	0.87	1.15
	urban 10,000-100,000	0.79	1.27
	urban >500,000	0.85	1.18
Education level	secondary or post-secondary non-university	0.61	1.63
	university Masters'	0.64	1.57
	PhD	0.86	1.17
Daily Internet use	<1 hours	0.72	1.39
	1 to 2 hours	0.66	1.51
	> 2 to 4 hours	0.66	1.51
Daily social media use	<0.5 hours	0.63	1.59
	0.5 to 1	0.66	1.51
	> 2 hours	0.60	1.67

Table S3. EFA summary statistics (polychoric matrix)

**Statistic**	**Value**
Sample size (EFA)	367
KMO	0.909
Bartlett's test: χ^2(df)	1655.547 (66)
Bartlett's test: p	<.001
Eigenvalue 1	5.409
Variance explained by factor 1	45.1%
Eigenvalue 2	0.991
Communalities (range)	0.303–0.702
Interfactor correlation in 2-factor benchmark	|r|=0.665

Table S4. Polychoric correlation matrix for DCQfN items*

	**Item 1**	**Item 2**	**Item 3**	**Item 4**	**Item 5**	**Item 6**	**Item 7**	**Item 8**	**Item 9**	**Item 10**	**Item 11**	**Item 12**
**Item 1**	1.000	0.493	0.540	0.550	0.346	0.387	0.392	0.376	0.473	0.372	0.447	0.358
**Item 2**	0.493	1.000	0.609	0.413	0.293	0.323	0.453	0.403	0.293	0.263	0.302	0.289
**Item 3**	0.540	0.609	1.000	0.444	0.389	0.393	0.509	0.439	0.312	0.300	0.357	0.386
**Item 4**	0.550	0.413	0.444	1.000	0.403	0.535	0.513	0.475	0.468	0.420	0.407	0.356
**Item 5**	0.346	0.293	0.389	0.403	1.000	0.484	0.334	0.345	0.373	0.262	0.276	0.361
**Item 6**	0.387	0.323	0.393	0.535	0.484	1.000	0.421	0.394	0.366	0.333	0.432	0.327
**Item 7**	0.392	0.453	0.509	0.513	0.334	0.421	1.000	0.473	0.470	0.384	0.438	0.394
**Item 8**	0.376	0.403	0.439	0.475	0.345	0.394	0.473	1.000	0.440	0.405	0.422	0.421
**Item 9**	0.473	0.293	0.312	0.468	0.373	0.366	0.470	0.440	1.000	0.413	0.373	0.314
**Item 10**	0.372	0.263	0.300	0.420	0.262	0.333	0.384	0.405	0.413	1.000	0.412	0.342
**Item 11**	0.447	0.302	0.357	0.407	0.276	0.432	0.438	0.422	0.373	0.412	1.000	0.399
**Item 12**	0.358	0.289	0.386	0.356	0.361	0.327	0.394	0.421	0.314	0.342	0.399	1.000

* Polychoric correlations were estimated from ordinal item responses. The diagonal equals 1 by definition.

Table S5. Eigenvalues and variance explained (polychoric matrix)*

Factor	Eigenvalue	% variance	Cumulative %	Extraction SS loadings	Extraction %	Extraction cumulative %
1	5.409	45.075	45.075	4.873	40.609	40.609
2	0.991	8.257	53.332	0.538	4.483	45.092
3	0.850	7.081	60.413			
4	0.761	6.343	66.756			
5	0.664	5.533	72.289			
6	0.635	5.289	77.579			
7	0.580	4.832	82.411			
8	0.521	4.342	86.753			
9	0.512	4.267	91.020			
10	0.402	3.352	94.372			
11	0.379	3.154	97.526			
12	0.297	2.474	100.000			

*Only the first factor exceeded 1 (Kaiser criterion). Extraction statistics are shown for the two-factor benchmark.

Table S6. Communalities (principal axis factoring on polychoric matrix)

**Item**	**Initial**	**Extraction**
**Item 1**	0.501	0.479
**Item 2**	0.437	0.558
**Item 3**	0.521	0.702
**Item 4**	0.506	0.535
**Item 5**	0.333	0.303
**Item 6**	0.420	0.414
**Item 7**	0.466	0.477
**Item 8**	0.410	0.440
**Item 9**	0.402	0.429
**Item 10**	0.309	0.359
**Item 11**	0.377	0.399
**Item 12**	0.309	0.316

* Extraction communalities quantify the proportion of each item's variance accounted for by the extracted common factors.

Table S7. Oblique two-factor benchmark solution: structure matrix and derived pattern matrix.

**Item**	**Structure: Factor 1**	**Structure: Factor 2**	**Pattern (derived): Factor 1**	**Pattern (derived): Factor 2**
**Item 1**	0.648	-0.612	0.432	-0.325
**Item_2**	0.514	-0.747	0.031	-0.726
**Item_3**	0.592	-0.837	0.063	-0.795
**Item_4**	0.728	-0.536	0.666	-0.093
**Item_5**	0.546	-0.411	0.489	-0.086
**Item_6**	0.643	-0.438	0.631	-0.019
**Item_7**	0.673	-0.563	0.535	-0.207
**Item_8**	0.659	-0.493	0.594	-0.098

**Item_9**	0.652	-0.381	0.715	0.094
**Item_10**	0.593	-0.330	0.670	0.115
**Item_11**	0.632	-0.404	0.651	0.029
**Item_12**	0.560	-0.411	0.514	-0.069

* Structure entries are item–factor correlations. Pattern entries are regression coefficients obtained as Pattern = Structure × Φ^{-1}.

Table S8. Standardized loading for the 1-factor, correlated 2-factor, and bifactor CFA models

Item	Loading on general factor	Loading Factor 1: Attitudes	Loading Factor 2: Knowledge/ Skills	Bifactor general	Bifactor Attitudes	Bifactor Knowledge/ Skills	IECV
Item 1	0.635	0.653		0.639	0.199		0.912
Item_2	0.666	0.683		0.658	0.274		0.853
Item_3	0.712	0.732		0.714	0.398		0.763
Item_4	0.686	0.704		0.709	-0.060		0.993
Item_5	0.527	0.541		0.568	-0.349		0.725
Item_6	0.621	0.639		0.653	-0.185		0.926
Item_7	0.604		0.623	0.579		0.177	0.915
Item_8	0.638		0.658	0.582		0.309	0.781
Item_9	0.653		0.679	0.593		0.361	0.730
Item_10	0.632		0.654	0.575		0.325	0.758
Item_11	0.601		0.621	0.538		0.348	0.705
Item_12	0.574		0.593	0.538		0.225	0.851

IECV - item explained common variance attributable to the general factor. Negative residual loadings on the bifactor Attitudes-specific factor indicate weak and unstable residual variance after accounting for the general factor

Table S9. Scaled/robust fit indices for the 1-factor, correlated 2-factor, and bifactor CFA models

Model	Scaled χ^2 (df)	p	Robust CFI	Robust TLI	Robust RMSEA (90% CI) / SRMR
1-factor	172.492 (54)	<.001	0.922	0.905	0.078 (0.065–0.091); 0.047
2-factor	141.316 (53)	<.001	0.938	0.923	0.070 (0.057–0.083); 0.043
Bifactor	75.195 (42)	0.001	0.969	0.951	0.056 (0.039–0.072); 0.031

Table S10. Ancillary bifactor indices for the DCQfN bifactor CFA model

Index	Value
Omega total (ω)	0.898

Omega hierarchical (ωH)	0.849
Explained common variance (ECV), general factor	0.824
Explained common variance (ECV), Attitudes-specific factor	0.079
Explained common variance (ECV), Knowledge/Skills-specific factor	0.097
Percent of uncontaminated correlations (PUC)	0.545
Model converged	Yes
Negative residual variances detected	No
Non-significant standardized loading in Attitudes-specific factor	Item 4
Negative standardized loadings in Attitudes-specific factor	Items 5 and 6
Range of standardized loadings, Knowledge/Skills-specific factor	0.177–0.361

Table S11. Measurement invariance of the final one-factor DCQfN model across age tertiles

Model	χ^2	df	CFI	TLI	RMSEA	SRMR	$\Delta\chi^2$	Δdf	p
Configural	465.368	162	0.944	0.932	0.084	0.058	—	—	—
Thresholds	487.572	232	0.953	0.960	0.064	0.058	54.744	70	0.910
Thresholds + loadings	465.154	254	0.961	0.970	0.056	0.059	23.774	22	0.359

Table S12. Sensitivity analysis for the linear regression model predicting DCQfN score after exclusion of 11 observations with deleted studentized residuals outside the ±3 threshold.

Variable	Category	B (SE)	ß	95%CI	p
e-Health literacy		0.70 (0.04)	0.49	0.62 - 0.79	<0.001
The number of programs used at work		0.16 (0.10)	0.06	-0.02 - 0.35	0.088
Age (years)		-0.02 (0.02)	-0.04	-0.06 - 0.01	0.188
Sex	female#				
	male	-2.10 (0.96)	-0.07	-3.98 - -0.22	0.028
Specialization	no#				
	yes	-0.63 (0.39)	-0.05	-1.41 - 0.14	0.107
Hospital employment	no#				
	yes	-0.32 (0.45)	-0.03	-1.20 - 0.56	0.473
Employment in a private facility	no#				
	yes	-0.09 (0.41)	-0.01	-0.90 - 0.72	0.823
Training recency	last 12 months#				
	>5 years ago	-0.29 (0.66)	-0.01	-1.58 - 1.01	0.666
	2-5 years ago	-0.36 (0.51)	-0.02	-1.37 - 0.64	0.478
	13-24 months ago	-0.23 (0.51)	-0.01	-1.23 - 0.76	0.644
Place of residence	urban 100,000-500,000#				
	rural	-0.25 (0.61)	-0.01	-1.44 - 0.95	0.686
	urban <10,000	0.08 (0.75)	0.00	-1.40 - 1.55	0.917
	urban 10,000-100,000	-0.27 (0.45)	-0.02	-1.16 - 0.62	0.551
	urban >500,000	-0.17 (0.60)	-0.01	-1.35 - 1.00	0.774
Education level	university Bachelors'#				

Variable	Category	B (SE)	ß	95%CI	p
	secondary or post-secondary non-university	-0.11 (0.59)	-0.01	-1.27 - 1.05	0.851
	university Masters'	0.36 (0.46)	0.03	-0.54 - 1.26	0.436
	PhD	0.32 (0.93)	0.01	-1.51 - 2.15	0.729
Daily Internet use	>4 hours#				
	<1 hours	-2.92 (0.64)	-0.16	-4.17 - -1.68	<0.001
	1 to 2 hours	-2.05 (0.55)	-0.14	-3.14 - -0.97	<0.001
	> 2 to 4 hours	-1.25 (0.47)	-0.10	-2.17 - -0.33	0.008
Daily social media use	1 to 2 hours#				
	<0.5 hours	-0.52 (0.57)	-0.04	-1.63 - 0.59	0.360
	0.5 to 1	-0.24 (0.54)	-0.02	-1.30 - 0.83	0.664
	> 2 hours	-0.87 (0.50)	-0.07	-1.86 - 0.11	0.083

# - reference category

Figure S1. Scree plot for the DCQfN based on the polychoric correlation matrix

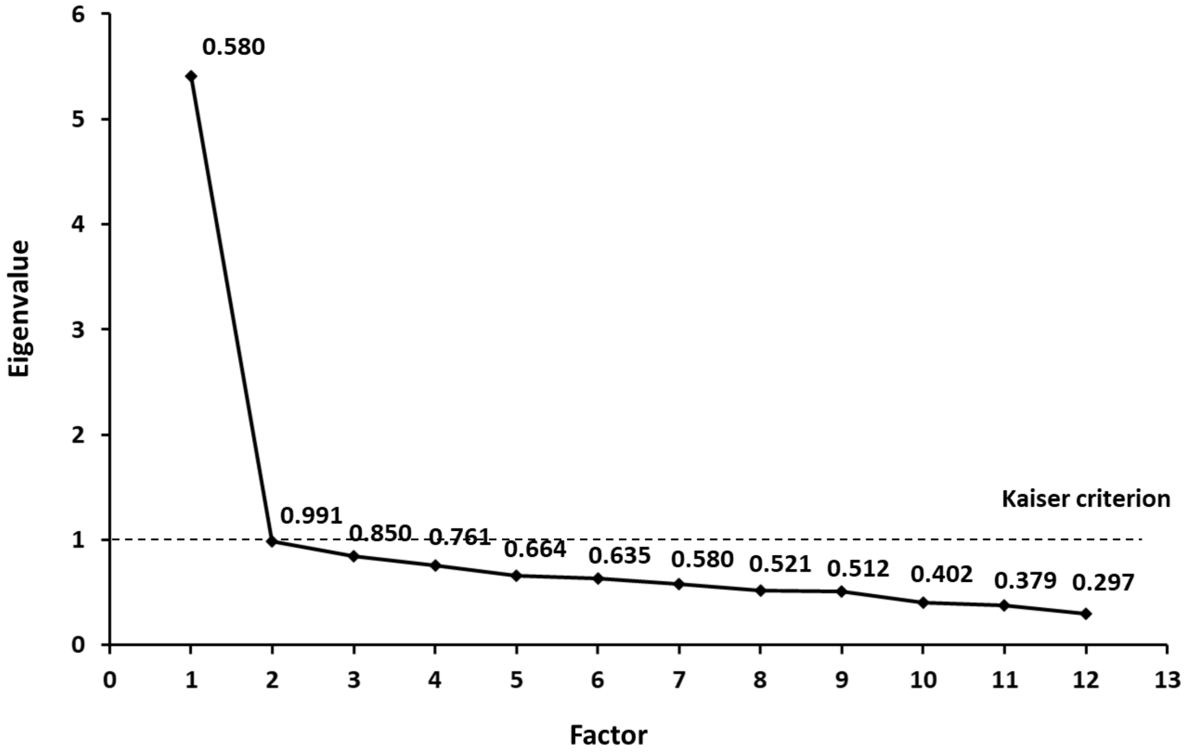

Note: Eigenvalues are from the exploratory factor analysis of the polychoric correlation matrix. The dashed horizontal line indicates the Kaiser criterion of eigenvalue >1.0. The sharp decline after the first factor and the fact that only the first eigenvalue exceeded 1.0 support a predominantly one-factor structure. Abbreviations: DCQfN - Digital Competence Questionnaire for Nurses.

Figure S2. One-factor confirmatory factor analysis model for the DCQfN

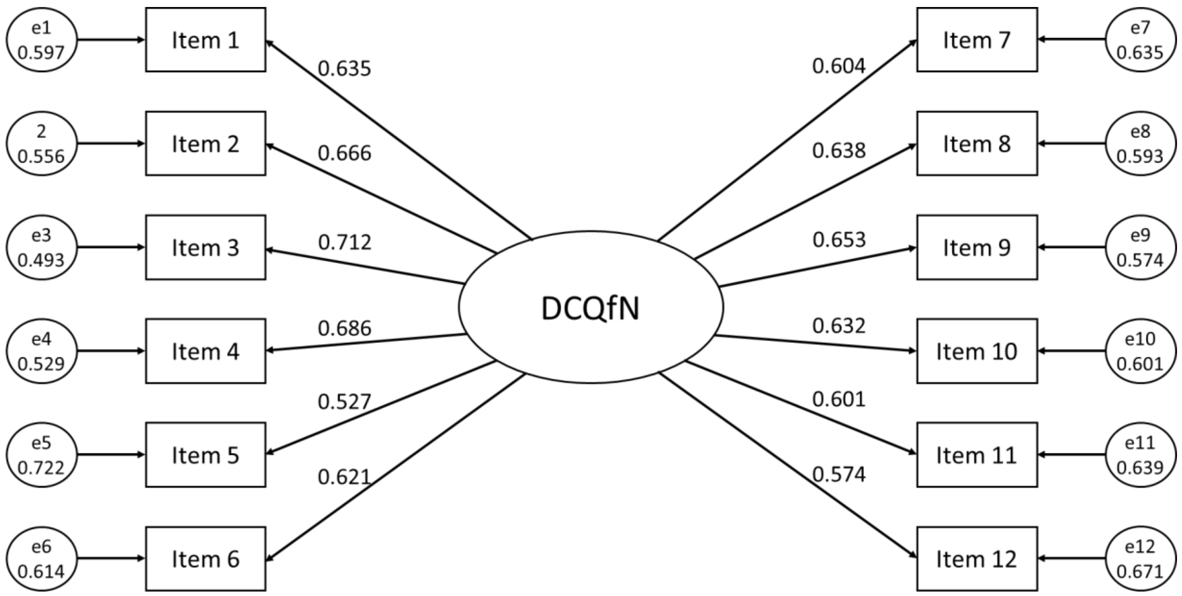

Note: Values on arrows are standardized factor loadings. Values in error circles indicate standardized residual variances, that is, the proportion of item variance not explained by the latent factor. Model fit: scaled $\chi^2(54)=172.492$, $p<.001$, robust CFI=0.922, robust TLI=0.905, robust RMSEA=0.078, 90%CI 0.065–0.091, SRMR=0.047. Model parameters are based on the standardized solution for the one-factor CFA model. Abbreviations: DCQfN - Digital Competence Questionnaire for Nurses.
